# Primary Angiosarcoma of the Thyroid in an Asian Woman: A Case Report with Review of the Literature

**DOI:** 10.1155/2020/9068506

**Published:** 2020-03-31

**Authors:** Shih-Hsuan Huang, Shang-Chung Wu

**Affiliations:** ^1^Department of Pathology, Kuang Tien General Hospital, Dajia, No. 321, Jingguo Rd., Dajia Dist., Taichung City 437, Taiwan; ^2^Department of General Surgery, Kuang Tien General Hospital, Dajia, No. 321, Jingguo Rd., Dajia Dist., Taichung City 437, Taiwan

## Abstract

Primary thyroid angiosarcoma is a rare malignant tumor and characterized by its prevalence in Alpine regions of Central Europe, close relation to longstanding goiter, and aggressive clinical course with dismal prognosis. We describe the case of an 83-year-old Chinese female who lives in the coastal area of Taiwan. She came to our hospital due to a progressively enlarged mass at her anterior neck. The sonography of the thyroid revealed a well-circumscribed mass in the left lobe. She underwent left hemithyroidectomy. The diagnosis of angiosarcoma of the thyroid was made and further confirmed at a different institution. To the best of our knowledge, this is the first case of primary thyroid angiosarcoma reported from Taiwan and the sixth Asian afflicted with primary thyroid angiosarcomas in the English literatures. The literature search in the PubMed database identified 58 cases who had histologically proven primary thyroid angiosarcomas. A preliminary analysis of epidemiological presentation, clinical features, immunohistochemical characters, and prognosis of primary thyroid angiosarcoma was proposed. The prognosis of this rare neoplasm is surprisingly favorable in comparison with that of its morphological similar, the anaplastic thyroid carcinoma. The use of a comprehensive panel of immunohistochemical stains, including at least two endothelial markers (especially CD31 and ERG) and PAX-8, in combination with thorough light microscopic examination may assist in the discrimination between these two tumors.

## 1. Introduction

Primary sarcomas are rarely seen in the thyroid gland, and the reported frequency ranges from 0.01% to 1.5% [1]. Among all histologic types, angiosarcoma is the most common mesenchymal malignancy, followed by malignant hemangioendothelioma [1]. Primary thyroid angiosarcoma (TAS) was originally reported in patients who resided in the Alpine countries of Central Europe where TAS comprises 2-10% of all thyroid malignancies in different series [2, 3]. Only sporadic cases have been described in non-Alpine areas [2]. Its predilection in these endemic goitrous regions and frequent coexistence with multinodular goiters suggest that the dietary iodine deficiency may be a potential cause [2]. Possible associations with exposure to vinyl chloride and radiation have been also proposed in the literatures [4–6]. TAS affects most often the elderly patients, especially in the seventh decade of life, with a female predominance (the female-to-male ratio is 4.5 : 1) [2]. TAS has a tendency to early metastasis at regional lymph nodes, lung, skin, bones, and soft tissues [6]. The overall prognosis is dismal, and most patients die from disease in less than 6 months [2]. We herein present a case of TAS diagnosed in an 83-year-old woman who lives in the coastal area of Taiwan and is alive at 66 months after surgery. To the best of our knowledge, this is the first case reported from Taiwan and the sixth Asian afflicted with TAS in the English literatures [5, 7–10].

## 2. Case Presentation

**Figure 1:**
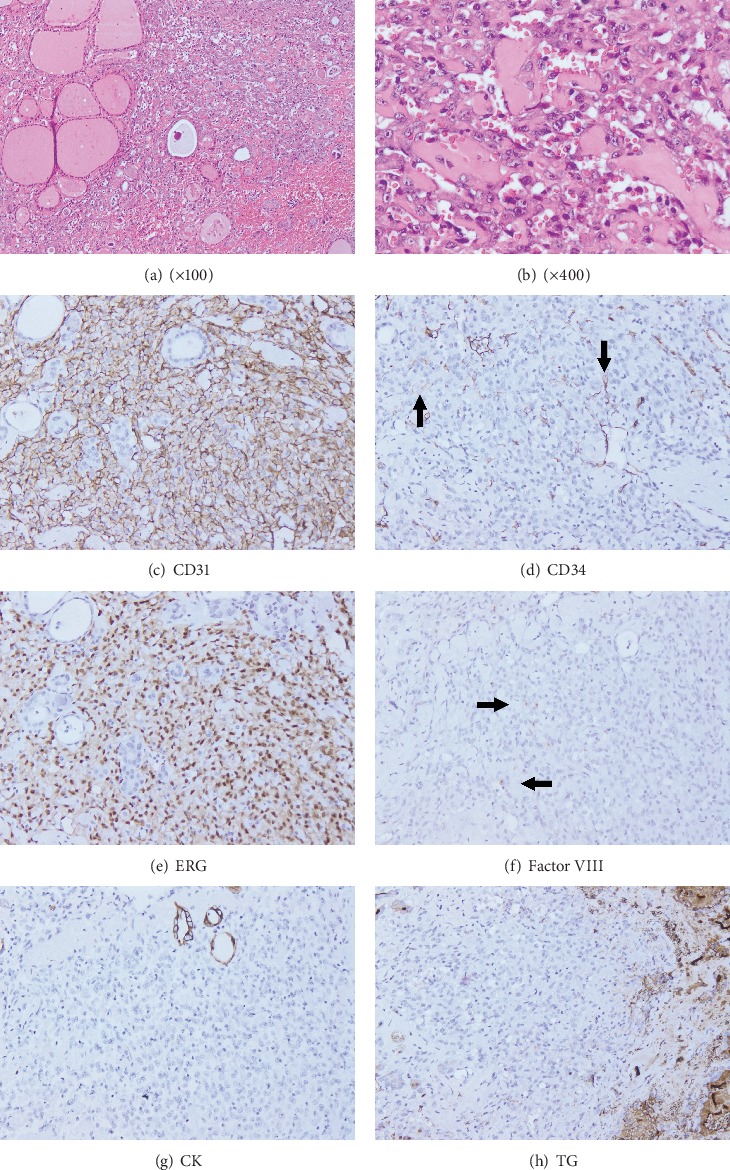
(a) The tumor is hemorrhagic and composed of ramifying and intercommunicating vascular spaces with infiltration into adjacent thyroid tissue (Mag.100x). (b) Those tumor cells have large epithelioid and pleomorphic nuclei with clumped chromatin and prominent basophilic nucleoli, and abundant eosinophilic cytoplasm exhibiting occasional intracytoplasmic lumina with identifiable erythrocytes (Mag.400x). (c) Diffuse membranous and cytoplasmic immunoreactivity for CD31 (Mag.200x). (d) Faint and focal expression of CD34 (Mag.200x). (e) Diffuse nuclear staining for ERG (Mag.200x). (f) Faint and focal cytoplasmic immunoreactivity for Factor VIII-associated antigen (Mag.200x). (g) Negative staining for pancytokeratin in lesional cells (Mag.200x). (h) No expression for thyroglobulin (Mag.200x).

An 83-year-old female without past history of major systemic disease had a progressively enlarged mass at her anterior neck accompanied by difficulty in swallowing for several months. Physical examination showed a movable mass at the left anterior neck. The sonography of thyroid revealed multiple variable-sized nodules in bilateral lobes; the largest one was a mixed hypoechoic and isoechoic lesion with circumscribed border and peripheral halo, about 3.7 cm in greatest dimension, in the left lobe. The levels of T3 and T4 were within normal limits, but elevated TSH level with hypocalcemia was noted. The fine needle aspiration was not performed. Chest radiography showed two small spherical calcified foci (up to 0.6 cm in greatest dimension), in favor of granulomas, in the right lower lung field. Under the impression of multinodular goiter, she underwent left hemithyroidectomy. On gross examination, the tumor measured 2.5 × 2.3 × 1.0 cm and was well-demarcated and partially circumscribed by a thick fibrous pseudocapsule. It revealed cystic change with brown friable contents and tan gray soft to rubbery areas around the cystic space. Microscopically, the tumor was located beside blood clots and cell debris with infiltration into non-neoplastic thyroid tissue (Figure 1(a)) and fibrous pseudocapsule. It comprised ramifying and anastomosing channels and sheet-like cellular growth. Those tumor cells were large, harboring abundant eosinophilic cytoplasm and round, irregular and pleomorphic nuclei with clumped chromatin and prominent basophilic nucleoli (Figure 1(b)). Some cells exhibited intracytoplasmic lumina containing identifiable red blood cells (Figure 1(b)). Frequent mitoses were found. The remaining thyroid tissue was limited and composed of variable-sized follicles which were filled with colloids and showed oncocytic change. Immunohistochemically, the tumor cells strongly and diffusely expressed CD31 (Figure 1(c)) and ERG (Figure 1(e)), but only faintly and focally stained by CD34 (Figure 1(d)) and Factor VIII-associated antigen (Figure 1(f)). All the epithelial markers, including CK (Figure 1(g)), EMA, and CAM5.2, and thyroglobulin (Figure 1(h)) were negative. The findings of light microscopy and immunohistochemical studies corroborated the diagnosis of angiosarcoma. The diagnosis was further confirmed at a different institution. The patient was lost to follow-up after discharge. We contacted her for informed consent of publication and found that this patient was alive but refused further evaluation.

## 3. Discussion

Primary angiosarcoma of thyroid gland (TAS) was originally described by Swiss authors as a separate type of neoplasm of endothelial origin in the late 19th and early 20th centuries [11]. It was firstly reported in the English literatures and termed as “hemangioendothelioma” by American authors in 1931 [12]. The use of immunohistochemical techniques and electron microscopy helped Chan and coworkers verify the endothelial cell origin of this tumor and present the first documented case who was not a Caucasian [7]. Eusebi et al. interpreted such tumors as “keratin-positive epithelioid angiosarcoma” since they exhibited strong immunoreactivity for keratin in addition to solid features of endothelial cell differentiation [13]. But some authors considered that these angiomatoid thyroid neoplasms exhibiting concomitant expression of epithelial and endothelial differentiation were high-grade carcinomas displaying variable mesenchymal (endothelial) metaplasia [14]. Papotti et al. stated that thyroglobulin messenger RNA which was detected at low levels in anaplastic carcinoma of thyroid was not found in angiosarcoma [15, 16]. Moreover, Kuhn et al. defined angiosarcoma and anaplastic carcinoma of the thyroid as two distinct entities based on considerable differences in genetic alterations in a recent study; the TP53 somatic mutations and TERT promoter mutations which are frequently identified in anaplastic carcinoma are not found in angiosarcoma [17].

A literature search, performed in April 2019 and using the keywords of “angiosarcoma” and “thyroid”, in the PubMed database revealed 58 cases who had histologically confirmed TAS [3–11, 13, 14, 16, 18–38], and our case was added to make a database of 59 cases. Those articles which were written in languages other than English and lacked available full texts and details of individual patients were eliminated.

Epidemiologically, only 4 cases were from the Alpine region (2 from Slovenia and 2 from France) [19, 38]. The residences were not specified in 5 cases [14, 24]. The vast majority (50 cases, 84.7%) of the included cases were from the non-Alpine region. Most patients were from the flatlands or coastal areas of Italy and Czech/Slovak Republic, 20 and 8 cases out of 50, respectively. The others were from United States of America (5 cases), Turkey (3 cases), the Republic of Korea (2 cases), Portugal (2 cases), China (Hong Kong SAR), Netherlands, Belgium, Brazil, Singapore, Malaysia, Iran, Canada, Romania, and Taiwan. The low number of documented cases from the Alpine region in the database could be due to a lack of rarity (relatively high incidence) in this area [3]. Besides, many articles were written in German, French, and Italian and not enclosed in this database. Except for 1 Negroid patient (Liberian) and 6 Asian patients, all the patients afflicted with TASs were white. Sex of one patient was not mentioned [14]. Thirty-five (60.3%) patients were female; a female predominance with a male to female ratio of 1 : 1.52 was noticed. The age at diagnosis ranges from 21 to 89 years (mean age 65.2) with the peak incidence in the seventh decade of life; forty-four (75.9%) patients were more than 60 years of age. The age of patient was not specified in three cases [14, 16, 34]. These data were similar to those of the previous case series [30].

Twenty-seven (45.8%) patients had concurrent and histologically proven goiter or a known history of goiter. Other coexistent pathological findings were Hurthle cell adenoma, 1 patient [13]; minimally invasive follicular carcinoma, 1 patient [8]; papillary carcinoma, 2 patients [24, 32]; and Hashimoto's thyroiditis, 2 patients [10, 28]. Two patients harbored more than one mass [11]. The etiology of TAS remains unclear. The most widely accepted hypothesis is that repeated intranodular hemorrhage and infarction in a longstanding nodular goiter with recurrent neovascularization result in subsequent malignant transformation of endothelial cells [16, 20, 27–29, 38]. Patients exposed to well-known predisposing factors for angiosarcomas of the skin, soft tissue, and liver, such as radiation and vinyl chloride, have been sporadically reported [4–6]. The lack of the above risk factors in half of the patients in this database and the frequent occurrences in Europe (38 out of 55 cases), especially in Italy, imply the possible role of other environmental agents in the pathogenesis of TAS.

The size of tumor was variable, ranging from 2.5 to 14.7 cm in its greatest dimension. Most tumors were ≧5 cm (Table 1). The details about extrathyroid extension, local recurrences, and distant metastases were listed in Table 1. Immunohistochemical stains for endothelial and epithelial markers were administered in all cases except for one [26] (Table 2). It is difficult to distinguish between TASs and anaplastic carcinomas with angiomatoid appearance due to the frequent expression of cytokeratin in the former. Moreover, both tumors are not immunoreactive for thyroglobulin and TTF-1 IHC stains [39] (Table 2). Therefore, the identification of endothelial differentiation in a high-grade neoplasm with angiomatoid features is very crucial. Antibody against CD31 is highly restricted to endothelial neoplasms, being expressed in more than 90% of angiosarcomas [40]. ERG, another currently available endothelial marker, has been reported to be expressed in 96% of angiosarcomas and very rare epithelial neoplasms [40]. It is worth noting that nuclear expression of ERG has also been observed in blastic extramedullary myeloid tumors, various cartilaginous tumors, approximately 50% of prostatic adenocarcinomas, and about 5% to 10% of epithelioid sarcomas [40]. The nuclear expression and generally excellent specificity for vascular tumors make ERG a useful adjuvant to the diagnoses of vascular neoplasms. The specificity for vascular neoplasms of FLI-1 is variable in different studies [40]. Although the studies have had discrepant results of the PAX-8 expression in anaplastic thyroid carcinomas, PAX-8 is found in 79% of anaplastic thyroid carcinomas in some research [39, 41] and may be a potentially useful tool for discriminating anaplastic thyroid carcinomas from other high-grade neoplasms in the thyroid gland. Thorough light microscopic examination in combination with a comprehensive panel of immunohistochemical stains, including at least two endothelial markers (especially CD31 and ERG) and PAX-8, could be of help in the workup of epithelioid neoplasms with angiomatoid morphology in thyroid glands.

The treatment strategies for TASs are diverse. Complete surgical resection with a clear margin is the primary mode of therapy, and neoadjuvant or adjuvant radiotherapy and/or chemotherapy may be beneficial to systemic and local disease control [16].

Among patients with documented follow-up (49 cases in total), twenty-eight patients died of disease because of postoperative complications, local recurrences, or distant metastases (Table 3), with the time of death ranging from 7 days to 36 months following the diagnosis. Most of them (25 cases, 89.3%) succumbed within 9 months. Sixteen patients (32.7%) were alive without disease; the follow-up periods ranged from 15 to 82 months after the diagnosis (Table 4). Nine patients (18.4%) remained disease-free for more than 36 months. Patients who were alive without evidence of disease in this database display variable conditions of age, tumor size, extrathyroidal extension, and lymph node metastasis (Table 4). But distant metastasis is found or mentioned in none of them. It is reasonable to speculate that distant metastasis may be the most relevant prognostic factor to long-term disease-free survival. Three patients who had lymph node metastasis at presentation or postsurgical local recurrence and received further surgery or adjuvant radiochemotherapy were alive without evidence of disease for at least 16 to 51 months after the adjuvant treatments (Table 4; [4, 28, 33]). Locoregional lymph node metastasis, a not uncommon finding in soft tissue angiosarcomas [42], was found in 8 cases (14.3%) of our database. Some authors claimed that patients of soft tissue angiosarcomas with isolated lymph node metastasis and treated intensively with multimodal therapy showed somewhat better outcomes, approaching those of patients with localized high-risk disease [42]. Therefore, a sentinel lymph node biopsy should also be considered as part of the surgical treatment in patients who have TASs but do not have radiologically identified metastatic disease. It is possible that a therapeutic lymph node dissection combined with multimodality therapy can improve disease free survival in patients of TASs with pathologically confirmed lymph node metastases.

## 4. Conclusion

Even though TAS is extremely rare in non-Alpine regions, it should be listed in the differential diagnoses of high-grade epithelioid neoplasm of thyroid. Nearly one-fifth of patients in our database stay alive and disease free for more than 3 years. The potentially prognostic differences between TAS and its more commonly encountered similar, anaplastic thyroid carcinoma, highlight the importance of precise diagnosis. A comprehensive panel of immunohistochemical stains, including at least two endothelial markers (especially CD31 and ERG) and PAX-8, could be of help in the diagnostic workup, and the application of ultrastructural and molecular studies should be considered in ambiguous cases. The elimination of articles written in German, French, and Italian could exclude many cases from the Alpine region and make our database be close to the collection of non-Alpine TASs. The statistics of IHC stains are obviously flawed due to inconsistent methods, antibodies, and observers. The survival may be overestimated because of the relatively limited follow-up durations in some reports. After all, this article encloses a small number of literatures which document variable clinical information, heterogeneous treatment approaches and regimens, and inconsistent follow-up data of patients with TASs. It merely offers the observations and speculations on the treatment and prognosis. Further studies are essential to clarify the etiology, therapeutic strategy, and overall prognosis of this rare neoplasm.

## Figures and Tables

**Table 1:** Clinical features of enclosed patients (59 cases).

Tumor characteristics	Case number (%)
Size of tumor	
<5 cm	13 (22.0%)
≧5 cm	33 (55.9%)
No specified	13 (22.0%)
Extrathyroidal extension	
Present	21 (35.6%)
Not identified/not mentioned	38 (64.4%)
Local recurrence	
Present	16 (27.1%)
Not identified/not mentioned	43 (72.9%)
Metastasis throughout the whole course	
Not found	33 (58.9%)
LNs (mediastinal, cervical, supraclavicular, mediastinal, and paratracheal)	8 (14.3%)
Lungs/pleura	17
Bone/bone marrow	4
Brain	3
Skin	1
Esophagus	1
N/A	3 (including our case)

**Table 2:** Immunohistochemical features.

Immunostain	Number of positive staining	Number of negative staining	Not performed
CD31	48 (100%)	0	11
CD34	21 (44.7%)	26	12
Factor VIII	42 (91.3%)	4	13
FLI-1	6 (100%)	0	53
ERG	4 (100%)	0	55
CK	30 (65.2%)	16	13
EMA	6 (18.8%)	26	27
CAM5.2	9 (75%)	3	47
TG	0	47 (100%)	12

**Table 3:** Outcome.

Dead	
Died of disease	28 (57.1%)
Died of unrelated causes	1
Alive	
With no evidence of disease	16 (32.7%)
With disease	4
Indeterminate status of disease	1 (our case)
Lost to F/U or not mentioned	9

**Table 4:** Clinicopathological features, treatment, and follow-up of 16 patients of primary thyroid angiosarcoma with no evidence of disease.

Author	# case	Gender, age, risk factors	Tumor size (mm)	Extrathyroid extension at diagnosis	LN metastasis at diagnosis	Distant metastasis at diagnosis	Neoadjuvant therapy	Surgery	Adjuvant therapy	Follow-up status
Eusebi et al. [13]	#4	F, 61 y/o	40	N/A	N/A	N/A	None	Lobectomy	None	24 months
Lamovec et al. [19]	#1	F, 64 y/o, goiter	40	No	No	No	No	L't total and R't subtotal lobectomy	R/T	36 months
Maiorana et al. [11]	#1	F, 84 y/o, goiter	30	No	N/A	No	None	TT	None	66 months
	#3	F, 69 y/o, goiter	50	No	N/A	No	None	TT	None	32 months
	#4	F, 60 y/o, goiter	3 masses; up to 25	No	N/A	No	None	TT	None	27 months
Ryska et al. [3]	#3	F, 60 y/o	70	N/A	No	N/A	N/A	N/A	N/A	21 months
Innaro et al. [28]		F, 49 y/o, goiter	75	No	Yes	No	None	TT+neck LND	C/T	22 months totally (postoperative residual lymphadenopathy S/P adjuvant C/T; then NED for 16 months)
Altinay et al. [32]		F, 62 y/o, goiter	30	No	N/A	N/A	None	TT	None	15 months
Couto et al. [33]		F, 61 y/o	35	No	N/A	N/A	None	TT	R/T	72 months totally (2 months after surgery, locally recurred, S/P tumor excision+R/T, then NED for 48 months)
Yoon Moon et al. [5]		F, 48 y/o	N/A	Yes	N/A	N/A	None	TT+partial resection of the trachea	R/T	24 months
Collini et al. [4]	#3	M, 56 y/o	50	Yes	No	No	None	TT	C/T and R/T	82 months
	#4	F, 63 y/o	130	Yes	No	No	C/T	TT	C/T	70 months
	#5	M, 53 y/o	50	Yes	No	No	None	TT	C/T	59 months totally (8 months after surgery, locally recurred, S/P C/T+tumor excision), NED for 51 months, then lost to follow-up
Marina et al. [6]		M, 63 y/o, radiation exposure, goiter	120	No	No	No	None	TT+neck LND	C/T	62 months
Nechifor-Boilă et al. [38]	#1	F, 74 y/o, goiter	70	No	N/A	N/A	None	TT	None	40 months
	#2	M, 70 y/o, goiter	80	Yes	No	No	None	TT+neck LND	R/T	73 months

F: female; M: male; N/A: not applicable; C/T: chemotherapy; R/T: radiotherapy; TT: total thyroidectomy; NED: no evidence of disease.
